# Unexpected aflatoxin exposure in a woman in northern Italy: a case report

**DOI:** 10.4076/1757-1626-2-7736

**Published:** 2009-09-17

**Authors:** Riccardo Perduri, Stefania Gobba

**Affiliations:** 1 Department of Public Health Sciences, Postgraduate School of Occupational Medicine, Chair of Occupational Medicine, University of Modena and Reggio Emilia, Via Campi 287, 41125 Modena, Italy; 2 Department of Medical Oncology, Ospedale di Circolo e Fondazione Macchi, Viale Borri 57, 21100 Varese, Italy

## Abstract

**Introduction:**

In well-developed countries scant attention is currently devoted to the risk of unknown exposure to aflatoxins, highly toxic and carcinogenic compounds. Nevertheless, unexpected ingestion of foods contaminated by these toxins can occasionally occur virtually worldwide.

**Case presentation:**

A 38-year-old woman living in Northern Italy developed unexplained abdominal symptoms after ingestion of aflatoxin-contaminated meat. The possible relation between symptoms and aflatoxins ingestion, the potential risk of hepatocellular carcinoma and the medical management of the case are discussed.

**Conclusion:**

In well-developed countries aflatoxins exposure should be considered in case of unexplained abdominal symptoms. Cancer risk cannot be assessed, nevertheless individual counselling and periodical medical examinations are recommended.

## Introduction

Aflatoxins (AFs) are a group of difuranocoumarin derivatives produced by certain strains of *Aspergillus flavus* and *parasiticus.* There are 4 primary AFs, but aflatoxin B1 (AFB1), the most toxic, is usually predominant in foods [1].

*Aspergilli* are common in nature, and can colonize and contaminate various foods and feeds under favourable conditions of temperature and humidity [1].

AFs can also be found in the milk, liver and meat of animals which are fed with contaminated feed [2]-[4]. Additionally, AFs are quite stable during cooking [2,4], so decomposition is unlikely and absorption occurs also after consumption of cooked food.

Both in humans and animals exposure is related to consumption of contaminated food. Even if food contamination is more frequent in tropical countries, AFs can be found in a wide range of foods consumed in all world areas [5,6].

AFs are highly toxic in both animals and humans [1,5], and are included in Group 1 ("carcinogens for humans") by the International Agency for Research on Cancer (IARC), being considered among the most potent liver carcinogens. Nevertheless, cases of aflatoxicosis in humans have rarely been reported, especially in well developed countries. However the possibility of a large underdiagnosis is generally acknowledged, especially if few subjects are affected. A likely explanation is that medical services are less developed in those countries where high levels of AFs contamination in food occur while in well developed areas exposure is unexpected, and therefore cases may go unnoticed. The latter possibility is illustrated in the case report hereby presented.

## Case presentation

The subject is a 38-years-old woman living in Northern Italy. She smokes about 20 cigarettes per day and usually drinks 1-2 glasses of wine per day during meals. Her dietary habits are peculiar: since long time she has been having a marked preference for beef meat, while she does not like other types of meat or fish, nor vegetables or fruit. Accordingly her diet is substantially unbalanced, consisting mainly in grilled or roasted beef meat. As her family has long since had a cattle breeding, she is used to eating almost exclusively meat from the family farm.

In the winter of 2004 the family farm animals started to show various disorders, such as slowing growth rate, delayed sexual development, gastrointestinal tract disorders, increasing incidence of several infective diseases, abortions and increase of overall mortality. The veterinarians considered different causes and carried over various treatments, as antibiotic therapies, without significant results. Aflatoxicosis is a rare disease in the area, as a consequence the possibility of AFs intoxication was not considered at the time.

For years the animal feed had been supplied by the same producer, but in the late spring of 2007, among the attempts applied to find a solution, the feed supplier was changed.

In late summer of 2007 the cattle recovered and the disorders gradually subsided. Later on (autumn 2007) the patient's family acknowledged that some analyses performed on the animal feed back in 2006 had revealed AFB1 levels up to 25 µg/kg. The maximum level of AFB1 in animal feed permitted by the European Union (EU) is 5 µg/kg. Further data are missing, since the former supplier refused to produce records about other analyses in feed. Accordingly the possibility of higher concentrations of AFs in feed exists. After the finding of high AFB1 levels in feed, the symptoms and signs observed in the animals were reconsidered by the farm veterinarians, who agreed that an AFs intoxication was possible. Unfortunately it was not possible to perform specific laboratory tests in the animals since too much time had passed from the end of contaminated feed consumption. However a relation between the disorders observed in animals and AFs is supported by documented feed AFB1 levels exceeding the EU limit value, animal case history consistent with aflatoxicosis, temporal correspondence between onset of disorders and consumption of potentially contaminated feed, and submission of disorders after feed replacement, according to the temporality principle of causality.

In the subject under examination, previous medical history did not account for any relevant disorder nor hospitalization before winter of 2004, when episodes of nausea, abdominal swelling, constipation alternating with diarrhea and abdominal pain appeared. The frequency of symptoms increased during the following months including several episodes of intense abdominal pain.

The subject underwent several medical examinations to rule out major causes of abdominal pain. Repeated gastrointestinal X-rays and abdominal ultrasonography were performed from May to September 2005, along with a uterine ultrasound scan (November 2005), but they showed no apparent alterations. In September 2005 a stool examination was carried over and proved to be negative for pathological microorganisms or parasites, and no steatorrhea was observed. Antibody tests for celiac disease were negative. Repeated blood analyses were substantially normal, including pancreatic enzymes and liver function tests (serum glutamic-pyruvic and glutamic-oxaloacetic transaminases, alkaline phosphatase, gamma-glutamyl transpeptidase, bilirubin, albumin and total proteins). Blood viral hepatitis antibodies were negative.

During 2006-2007 abdominal pain was so intense that the patient repeatedly attended the Emergency Department of the local area hospital. Twice "mittelschmertz" was diagnosed, other times a generic diagnosis of "acute abdominal pain" was made. Tramadol was effective in pain reduction.

During the whole period the subject's diet remained substantially unmodified, and she has never ceased eating meet from the family farm. Nevertheless, after the animal feed supplier was changed in autumn of 2007, the meat was no more contaminated. The abdominal symptoms subsided by October 2007, and no further episodes of abdominal pain have ever since been reported.

The timing of signs and symptoms both in animals and in the case are described in Table 1.

**Table 1:** Time-line of symptoms and signs in the animals and in the case

## Discussion

The main target organ of AFs is the liver, and acute toxic hepatitis with high mortality rates have been reported [7]. In addition, in humans adverse effects in immune system [5], and gastrointestinal tract have been reported [8,9]. A role of AFs in Reye syndrome and in kwashiorkor has been assumed [1,10]. Other effects have been described, as an interference with metabolic processes and various essential micronutrients [1,5,10].

An assessment of the health risk possibly related to AFs exposure is difficult as the toxicokinetics in humans is complex and knowledge is incomplete [5]. In addition to intensity and duration of exposure, several factors, such as age and health conditions, seem to influence susceptibility to AFs effects [5].

Diet supposedly plays an important role as well [11]. One case of AFS acute intoxication in a well developed country has been described: a female laboratory worker in USA attempted suicide by repeatedly ingesting high doses of purified AFs. Except for transient rash, nausea and headache, she apparently had no adverse effects. Furthermore, in a 14-year follow-up she showed no signs of liver cancer [12]. This might suggest that in well-nourished persons AFB1 hepatotoxicity is lower than in experimental animals or in populations with poor nutrition, and that the growth latency of AFs-induced tumour may exceed 14 years.

Moreover, adverse effects related to AFs exposure can easily remain unrecognized by medical professionals, especially in well-developed countries. In fact diagnosis may be difficult, and frequently the demonstration of the role of AFs may be possibly based on likelihood criteria only, as in the case presented here.

In our patient it was impossible to measure AFs or their derivatives as exposure biomarkers [5] since the case came to our attention several months after interruption of the ingestion. Likewise, no reliable appraisal of the AFs ingested dose is possible. Nevertheless, the unbalanced diet, based on an excessive amount of beef meat, induced an unusually high exposure to these toxins, possibly furthering the onset of disorders.

The abdominal symptoms observed in our case are included among symptoms usually reported in AFs intoxication [9,10], and may be related to a direct effect of AFs in the colon, as described both *in vitro*[9] and in experimental animals [8]. The absence of other clinical adverse effects is consistent with knowledge about AFs exposure in well developed countries [12].

Causes of abdominal pain other than AFs were not identified despite repeated medical examinations and tests. No liver effects were observed performing blood analyses and ultrasound scans, but scientific literature has previously reported such findings in well nourished subjects [12].

The association between the patient's symptoms and AFs ingestion is therefore supported by several facts: i) the patient consumed meat from contaminated animals; ii) the clinical history is consistent with AFs intoxication, according to literature data; iii) a temporal correspondence is observed between the onset of disorders and the consumption of contaminated meat as well as between the withdrawal of symptoms and the stop of consumption; iv) no other causes were identified.

An open question is the risk of hepatocellular carcinoma (HCC). The carcinogenic activity of AFB1 is possibly related to its 8, 9-epoxide metabolite which can react with DNA. It can induce mutations in the codon 249 of the p53 gene [6,13]. The same mutations are described in HCC. The risk of HCC depends on different factors, among which the most important is hepatitis virus chronic infection. Two epidemiological studies accomplished in China showed that the relative risk of developing HCC was 3.4 in AFs exposed in relation to unexposed, and about 59 in case of both AFs exposure and positive HBsAg status [14]. The extrapolation of these data to EU population is difficult, nevertheless scientific evidence suggests that all doses may have a cumulative effect on the risk of developing cancer [5].

As to our case, the cancer risk assessment is impossible due to the lack of quantitative data about the ingested dose. Molecular cytogenetic tests were not performed in the patient. The diet of the patient was unbalanced, being poor in fruit and vegetables, which are known to reduce cancer risk [15].

As regards the medical management of the case, the use of chemopreventive agents as oltipraz or chlorophyllin, proposed in exposed subjects in endemic areas [15], is not recommended. Rather a vaccination is advised, as the subject is currently in a negative HBsAg status, and hepatitis B virus increases the risk of HCC. Furthermore, a periodical medical follow up is proposed. Restriction of alcohol intake and a more balanced diet are also recommended.

## Conclusion

The case hereby presented emphasizes the possibility of missing AFs exposure in well developed Countries, and suggests the need to consider an unnoticed AFs exposure in case of unexplained abdominal symptoms.

HCC risk related to AFs consumption cannot be usually assessed, nevertheless individual counselling for exposed people should take into account HBV vaccination, periodical medical examinations, alcohol restriction and a well-balanced diet.

## Consent

Written informed consent was obtained from the patient for publication of this case report. A copy of the written consent is available for review by the Editor-in-Chief of this journal.

## Competing interests

The authors declare that they have no competing interests.

## Authors' contributions

Both authors gave the same contribution to the manuscript.
